# Astrocytic Glutamate Transporter 1 (GLT1) Deficiency Reduces Anxiety- and Depression-Like Behaviors in Mice

**DOI:** 10.3389/fnbeh.2020.00057

**Published:** 2020-04-22

**Authors:** Yun-Fang Jia, Katheryn Wininger, Ada Man-Choi Ho, Lee Peyton, Matthew Baker, Doo-Sup Choi

**Affiliations:** ^1^Department of Molecular Pharmacology and Experimental Therapeutic, Mayo Clinic, Rochester, MN, United States; ^2^Neuroscience Program, Mayo Clinic, Rochester, MN, United States; ^3^Department of Psychiatry & Psychology, Mayo Clinic, Rochester, MN, United States

**Keywords:** glutamate transporter 1 (GLT1), anxiety, depression, astrocyte, behaviors, fear conditioning

## Abstract

Glutamatergic dysregulation is known to contribute to altered emotional regulation. Astrocytic glutamate transporter 1 (GLT1) is responsible for the majority of glutamate clearance from synapse. However, the role of astrocytic GLT1 in affective processes such as anxiety- and depression-like behavior is not fully understood. Here, we found that astrocytic GLT1 deficient mice entered more frequently, and spent more time in the open arms of elevated plus maze without difference in overall distance traveled in the open field, nor were there any metabolic changes observed in the metabolic chamber compared to wildtype mice. Moreover, mice lacking astrocytic GLT1 exhibited less immobile time and moved greater area in the tail suspension test. Similarly, in the forced swim test, they showed less immobile time and moved greater area. In addition, we found that astrocytic GLT1 deficiency reduced freezing responses in the fear contextual and cued tests. Taken together, our findings suggest that astrocytic GLT1 deficiency decreases anxiety and depression-like behaviors.

## Introduction

Major depressive disorder (MDD) is a mental health disorder that displays a combination of symptoms, including reduced motivation and activities, helplessness, loss of appetite or interest, and sleep disturbance (Kessler et al., 2003; Cui et al., 2014), causing significant impairment in daily life. Because of heterogeneity of depressive symptoms, it is difficult to identify molecular and cellular mechanisms underlying clinical symptoms. Generalized anxiety disorder (GAD) is one of the most common psychiatric disorders and co-occurring illness with MDD (Sunderland et al., 2010). This co-occurrence may indicate a similar etiology and pathophysiological abnormalities in MDD and GAD (Mineka et al., 1998). High rates of comorbidity (Zbozinek et al., 2012), and similar brain abnormalities are possibly attributed to both anhedonia and anxiogenesis (John et al., 2015). In addition, since the behavioral manifestations were similar between anxiety and fear, they may have overlapping neural basis of phenotypes (Tovote et al., 2015).

Glutamate homeostasis is critical for normal brain physiology. The precisely balanced control of glutamate release and uptake assure the physiological optimum (Kiryk et al., 2008). Glutamatergic dysregulation is known to be involved in psychiatric disorders including depression, anxiety, and fear-related disorders (Davis and Myers, 2002; Walker and Davis, 2002; Bergink et al., 2004; Cortese and Phan, 2005; Mathews et al., 2012; Murrough et al., 2017). In the central nervous system (CNS), extracellular and synaptic glutamate is regulated by a family of glutamate transporters, which removes glutamate from the synaptic cleft. Among five well-identified glutamate transporters, glutamate transporter 1 (GLT1) is responsible for the majority (90%) of glutamate clearance (Su et al., 2003).

Notably, multiple brain regions including the prefrontal frontal cortex, striatum, and hippocampus are important regulators of mood disorders. Abnormal gene expression and glial loss were found in a discrete region of postmortem prefrontal cortex of MDD (Kang et al., 2007; Di Benedetto et al., 2016; Ho et al., 2019). A significant decrease in total hippocampal volume was reported in recurrent or chronic MDD (Cobb et al., 2013). Moreover, GLT1 is differentially expressed across anatomical brain. For example, altered hippocampal GLT1 expression was found in rodent depression models (Pines et al., 1992; Blacker et al., 2019; Ho et al., 2019).

As GLT1 plays a critical role in glutamate homeostasis, mice deficient of GLT1 could elucidate the impact of glutamatergic disturbances. Global GLT1 null mice display excessive glutamate levels, and exhibit severe seizure activity including spontaneous seizures and increased susceptibility to acute cortical injury (Tanaka et al., 1997). Since astrocytes participate in the uptake, metabolism, and recycling of glutamate (Rajkowska and Miguel-Hidalgo, 2007), the loss of GLT1 in astrocytes may account for the alterations in glutamate neurotransmission in depression (Rajkowska and Stockmeier, 2013). In rodents, glial loss in the prefrontal cortex was demonstrated to be sufficient to induce depressive-like behaviors (Banasr and Duman, 2008). Post-mortem studies reported a loss of glial cell number in depressed patients, indicating together with abnormal functioning could contribute to the pathophysiology of mood disorders (Czeh and Di Benedetto, 2013; Di Benedetto et al., 2016). Notably, astrocytic-, but not neuronal-specific deletion of GLT1 induced fatal epilepsy, suggesting that astrocytic GLT1 performs critical functions (Petr et al., 2015). Further, recent data indicated that selective deletion of GLT1 in the diencephalon, brainstem and spinal cord was sufficient to reproduce the phenotypes (excess mortality, decreased body weight, and lethal spontaneous seizure) (Sugimoto et al., 2018). Deletion of GLT1 in habenula astrocytes increased neuronal excitability and depression-like behaviors (Cui et al., 2014). The blockade of GLT1 by administration of its inhibitor (DHK) in the central amygdala induced both depression and anxiety (John et al., 2015). In contrast, downregulation of GLT1 by administration with the DHK acutely increased glutamatergic neurotransmission, which triggers immediate antidepressant-like responses in rats (Gasull-Camos et al., 2017).

Overall, the contribution of GLT1 in regard to emotional regulation such as anxiety- and depressive-like behavior, as well as fear remains highly controversial. These studies together conclude that GLT1 exerts specific, and sometimes even opposite roles depending on cell type and regional specifically. Therefore, this study aimed to investigate the behavioral alterations of anxiety- and depression-like behaviors in astrocytic GLT1 deficient mice.

## Materials and Methods

### Animals

Mice were group-housed (4,5 animals per cage) in standard Plexiglas cages in a 12 h light/dark cycle (lights on at 6 AM and off at 6 PM) with a temperature (22–24°C) and humidity (50%) regulated environment with access to standard lab food and water ad libitum. The floxed-GLT1 mice (GLT1^F/F^) mice were obtained from Niels C. Danbolt’s laboratory (University of Oslo, Norway) (Gregorian et al., 2009) and the GFAP^cre/+^ line was purchased from the Jackson laboratory (Cat no., 024098 – B6.Cg-Tg(Gfap-cre)77.6Mvs/2J). To generate astrocyte-specific GLT1 knockout mice, we crossed the GLT1^F/F^ mice with the GFAP^cre/+^ line, in which Cre recombinase is expressed selectively in the astrocytes. All mice in this study had C57BL/6J genetic background. All animal care, handling procedures and experimental protocols were approved by the Mayo Clinic Institutional Animal Care and Use Committee (IACUC) in accordance with the guidelines set forth by the National Institutes of Health.

### Immunohistochemistry

Immunohistochemistry was done as previously described (Jia et al., 2019). Briefly, mice were euthanized by CO_2_ asphyxiation followed by rapid brain removal. Brains were fixed in 4% paraformaldehyde (Sigma Aldrich) for 24 h and then immersed in 30% (w/v) sucrose in 0.1 M PBS at 4°C for 48 h until the tissue sunk. Coronal sections (40 μm) were cut by a cryostat for the following brain regions: medial prefrontal cortex (mPFC), striatum, and hippocampus (Hip), and incubated as free-floating sections in 0.5% Triton-PBS for 15 min and then in 5% (w/v) bovine serum albumin (BSA)-PBS for 4 h at room temperature. Sections were then incubated with guinea pig anti-GLT1 primary antibody (1:2000, Cat No.: Ab1783, Millipore), or rabbit anti-GFAP primary antibody (1:500, Cat No.: Ab68428, Abcam), or rabbit anti-Iba1 primary antibody (1:500, Cat No.: Ab178846, Abcam), or rabbit anti-NeuN primary antibody (1:500, Cat No.: Ab177487, Abcam), at 4°C for overnight. After washing with PBS, slices were incubated with secondary antibody Alexa 568-conjugated goat anti-guinea pig (1:1000, Thermo Fischer Scientific), or Alexa 488-conjugated goat anti-rabbit (1:1000, Thermo Fischer Scientific), or Alexa 594-conjugated goat anti-rabbit (1:1000, Thermo Fischer Scientific) for 3 h at room temperature. Nuclei were visualized with DAPI (1:2000, sigma) added to the mounting solution. Images from the brain regions of interest were obtained on an LSM 510 confocal laser scanning microscope (Carl Zeiss).

### Stereotaxic Viral Injection

In order to visualize the GFAP-Cre positive cells, we purchased GFAP promoter driven Cre recombinase virus fused to eGFP (enhanced green fluorescence protein) tag from Vector Biolabs. A mixture of ketamine (100 mg/kg) and xylazine (10 mg/kg) were used for anesthesia. Eight-week old male C57BL/6J mice were positioned in a stereotaxic instrument (KOPF Instruments). A 35−gage syringe needle (World Precision Instruments) was used to deliver GFAP-Cre virus (AAV9−GFAP−Cre/eGFP; 1.4 × 10^13^ GC/mL, 1.0 μL, Vector Biolabs) into the NAc (AP: +1.3 mm, ML: +1.0 mm, DV: −4.25 mm) at a rate of 0.1 μL/min for 10 min. At the end of injection, needles remained in place for 5 min to ensure complete delivery of the viral bolus and were then slowly retracted to minimize trauma and viral spreading. The scalp was sutured with 5−0 polyviolene sutures (Sharpoint). To allow for sufficient viral expression, immunohistochemical staining began 3 weeks after surgery.

### Western Blot

Mice were euthanized by CO_2_ asphyxiation and rapidly decapitated. The mPFC, striatum, and Hip were immediately isolated under a surgical microscope. The extracted tissue was weighed and snap-frozen on dry ice for storage at −80°C until processing for WB. Each brain region was homogenized in a Storm 24 magnetic Bullet Blender for 4 min at a speed setting of 4 (Next Advance Inc., Averill Park NY, United States), with 0.5 mm zirconium oxide beads in combination with 50–70 μl of Cell-lytic MT mammalian tissue extraction reagent (Sigma-Aldrich) containing 50 mM Tris buffer (pH 7.4), 2 mM EDTA, 5 mM EGTA, and 0.1% SDS. The homogenization buffer contained Complete (Roche) protease inhibitor cocktail and phosphatase inhibitor cocktails Type II and III (Sigma-Aldrich). Homogenates were then centrifuged at 16,400 rpm (4°C) for 15 min and supernatants were collected for subsequent SDS PAGE and WB analysis. For western blot analysis, homogenates were loaded at 20 μg for all brain regions. Brain samples were separated on a 4–12% Nu-Page Bis-Tris gel in MOPS buffer (Invitrogen, Carlsbad, CA) at 80 V for about 1 h followed by 150 V for the remaining 1 h. This was followed by transfer to a PVDF membrane (Invitrogen) at 30 V for 1 h. Samples were blocked at room temperature for 1 h (5% BSA in 1× TBST), then immunoblotted overnight at 4°C (5% BSA in 1× TBST) with primary antibodies specific to GLT1 (1:1000; Millipore), GS (1:2000; Abcam), and GAPDH (1:2000; Millipore). Following three washes (1× TBST), immunoblots were incubated with respective anti-guinea pig, anti-rabbit, and anti-mouse secondary antibodies (1:2000, Millipore) for 1 h at room temperature. Blots were visualized with the Radiance Peroxide Substrate (Azure Biosystems, Dublin, CA, United States), developed on a Azure Image Station scanner (Azure Biosystems, Dublin, CA, United States), and band optical density quantification was performed using NIH ImageJ software.

### Quantitative Real-Time PCR (qRT-PCR)

Mice were euthanized by CO_2_ asphyxiation and rapidly decapitated. The mPFC, striatum, and Hip were immediately isolated under a surgical microscope. Total RNA was isolated using the RNeasy Plus Mini kit (Qiagen; Cat No. 74134) and then reverse-transcribed by the Life Technologies Superscript III First-Strand Synthesis SuperMix kit (Cat No. 18080400) to obtain cDNA. The thermal cycling protocol for reverse transcription was 30 min at 50°C followed by 15 min at 95°C. Quantitative RT-PCR was performed on CFX 96 Touch^TM^ Real-Time System, C1000 Touch Thermal Cycler (Bio-Rad) using QuantiTect SYBR Green RT-PCR Kit (Qiagen; Cat No. 204143) and gene-specific primers (GLT1, Gls, GS, xCT, and GAPDH; Qiagen). The thermal cycling protocol for qRT-PCR was 40 amplification quantification cycles of 15 s at 94°C, followed by 10 s at 55°C, and then 30 s at 72°C. The targeted gene mRNA expression was normalized to GAPDH. Percentage changes were calculated by subtracting GAPDH Ct values from Ct values for the gene of interest using the 2^–ΔΔ*Ct*^ method (Livak and Schmittgen, 2001).

### Behavioral Tests

Eight-week old male mice were used in the following behavioral observations. The behavioral experiments were performed between 9 AM and 5 PM. Mice were acclimated to the testing room for 30 min prior to each behavioral test. We used three independent batches of mice to avoid potential influences of behavioral tests while minimizing the use of animals. The batches are for (1) OFT, EPM, and metabolic chamber only, (2) TST and FST only, and (3) fear conditioning test. Within each batch, the behavioral tests were performed one week apart between tests.

### Open Field Test

The ENV-510 test environment equipped with infrared beams and Activity Monitor (Med Associates) were used to evaluate motor activity in the open field test (OFT). Mice were placed in a Plexiglas box (27 cm × 27 cm × 20.3 cm) and allowed to explore the chamber for 1 h. The data was recorded by each beam break as one unit of exploratory activity using the activity monitoring software (Med Associates).

### Elevated Plus Maze

Elevated plus maze (EPM) (Med Associates) was elevated 74 cm above the floor, which consisted of two open (35 cm × 6 cm) and two closed (35 cm × 6 cm × 22 cm) arms and a connecting central zone (6 cm × 6 cm) (Jia et al., 2014). Mice were placed in the center of the EPM, facing an open arm. Mice were allowed to freely explore the maze for 5 min. The amount of time spent in the open arms and the frequency of transitions between the open and closed arms were recorded and analyzed by the monitor software (Ethovision-XT, Noldus).

### Metabolic Chamber

Mice were placed in the metabolic cages (Oxylet Pro, PanLab) for 24 h for habituation, followed by 48 h to measure oxygen consumption and energy expenditure. Mice were maintained at a 12 h light/dark cycle with lights on at 6 AM and off at 6 PM. Mice were free to access food and water. Energy expenditure, vCO_2_, and vO_2_ were obtained using a gas analyzer (Panlab, LE 405 Gas Analyzer) and metabolism software (Panlab, Metabolism).

### Tail Suspension Test

Mice were suspended by the tail using adhesive Scotch tape, to a bar suspended 20 cm above the table (Jia et al., 2014). Individual mice were separated by a barrier and tests lasted for a total of 6 min. All sessions were video recorded. The total area moved, activity duration, and duration of time spent immobile were analyzed by Panlab Smart video tracking software (Harvard apparatus, Spain). Mice were considered immobile when they hung passively without any movement.

### Forced Swim Test

Each mouse was placed in a 2-liter beaker filled with water (depth = 30 cm; temperature 24–25°C) (Jia et al., 2014). Mice were forced to swim 6 min, and the time spent immobile during the last 4 min was video recorded. The total area moved, activity duration, and duration of time spent immobile were analyzed by Panlab Smart video tracking software (Harvard apparatus, Spain). They were considered immobile when they stopped struggling, only moved slightly and occasionally to keep their nose above the water surface.

### Fear Conditioning Test

The fear conditioning test was performed in four identical near infrared (NIR) Video Fear Conditioning Chambers (Med Associates, Fairfax, VT, United States), housed in a sound attenuating box. A speaker was located at the top of the right aluminum wall, and a house light at the top left aluminum wall. The floor grid was connected to an aversive stimulus. The behavior was recorded using a high speed firewire monochrome video camera with a near infrared pass filter on an 8 mm lens and analyzed using Video Freeze^®^ software (Med Associates). On day 1, mice were trained with 2 min of habituation to the chamber followed by 5 pairings of 30 s tone (75 dB, 3000 kHz) with a shock occurring the last 2 s of the tone (0.4 mA) and a 2-min inter-trial interval (ITI 2 min). On day 2 for the contextual test, mice were only presented the same context (no sound, no shock) used on day 1 for training for identical duration as day 1. On day 3 for the cued test, a white floor grid cover and a black A-frame chamber insert were added to the chamber. Mice were subjected to 2 min of habituation to the chamber followed by five presentations of 30 s tones (75 dB, 3000 kHz) without a shock at a 2-min inter-trial interval (ITI 2 min). Freezing response (%) was measured for all training and testing days by Video Freeze^®^ software (Med Associates). The chamber was cleaned with 70% ethanol and allowed to dry completely prior to testing in between each animal.

### Statistical Analysis

All data are expressed as mean ± SEM (standard error of the mean). Analyses were conducted using GraphPad Prism (version 6.0). Unpaired two-tailed Student’s *t*-test was used to compare the difference between two groups. Two-way repeated measures ANOVA was used to detect the effects of time and genotypes. ANOVA were followed by Tukey *post hoc* tests where interactions were found. Statistical significance was set at *p* < 0.05.

## Results

### GFAP-Positive Astrocyte-Specific Deletion of GLT1

**FIGURE 1:**
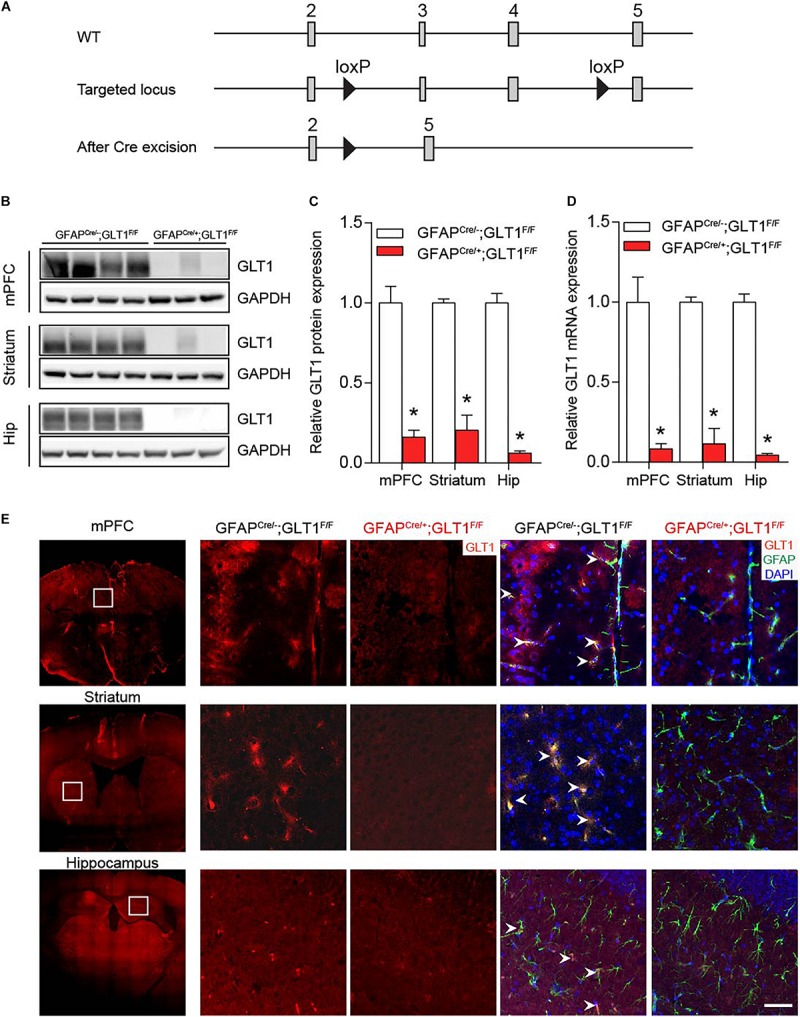
Ablation of GLT1 in astrocytes. **(A)** Generation of astrocytic- GFAP^cre/+^; GLT1^F/F^ mice. **(B)** Western blot analysis of GLT1 in the medialprefrontal cortex (mPFC), striatum, and Hippocampus (Hip). GLT1 band intensities were normalized with those of GAPDH. **(C)** The quantitative analysis of western blot of 1B (*n* = 3,4). **(D)** Quantitative real-time PCR analysis of GLT1 in the mPFC, striatum, and Hip (*n* = 3,4). GLT1 mRNA level were normalized with those of GAPDH. **(E)** GLT1 immunohistochemistry for three brain regions including the mPFC, striatum, and hippocampus. Scale bar = 20 μm. All data are presented as mean ± SEM. Student’s *t*-tests were used to compare GLT1 protein or mRNA levels of GFAP^cre/+^; GLT1^F/F^ mice and controls for each brain region. *p < 0.05.

We generated GFAP-positive astrocyte-specific GLT1 knockout mice (Figure 1A) by crossing the floxed-GLT1 mice (GLT1^F/F^) with the GFAP^cre/+^ line, in which Cre recombinase was expressed selectively in the astrocytes. Control mice had a genotype of GFAP^cre/–^; GLT1^F/F^.

Among multiple brain regions, preclinical and clinical studies have identified three main brain regions, prefrontal frontal cortex, striatum, and hippocampus, as cores of emotional regulation (Kang et al., 2007; Di Benedetto et al., 2016; Ho et al., 2019). Thus, we sought to examine the GLT1 mRNA and protein expression in these three brain regions. Both Western blot (two−tailed unpaired *t*-test; mPFC: *t*_5_ = 6.619, *p* = 0.001; Striatum: *t*_5_ = 9.264, *p* = 0.0002; Hip: *t*_5_ = 13.19, *p* < 0.0001; Figures 1B,C) and qRT-PCR (two−tailed unpaired *t*-test; mPFC: *t*_5_ = 4.890, *p* = 0.005; Striatum: *t*_5_ = 9.794, *p* = 0.0002; Hip: *t*_6_ = 18.52, *p* < 0.0001; Figure 1D) analyses of GLT1 revealed a significant reduction of GLT1 protein and mRNA in the medial prefrontal cortex (mPFC), striatum, and hippocampus (Hip) of GFAP^cre/+^; GLT1^F/F^ mice. Immunohistochemical analysis confirmed astrocytic GLT1 deletion in mPFC, striatum, and Hip of 8-week-old GFAP^cre/+^; GLT1^F/F^ mice (Figure 1E and Supplementary Figure S1). To validate the specificity of Cre expression in the GFAP positive astrocyte, we employed GFAP-Cre virus to visualize the co-localization with different cell-markers with eGFP markers since Cre recombinase itself is difficult to detect with antibodies. We injected GFAP-Cre viruses to C57BL6/J mice and analyzed co-localization with astrocytic (GFAP)-, microglia (Iba1)-, and neuronal (NeuN)-antibodies, respectively. Our results demonstrated that Cre expression is uniquely co-localized with GFAP antibody (Supplementary Figure S2A), without noticeable co-localization with either Iba1 (Supplementary Figure S2B) or NeuN antibodies (Supplementary Figure S2C), supporting the specificity of Cre expression in GFAP-positive astrocytes.

**FIGURE 2:**
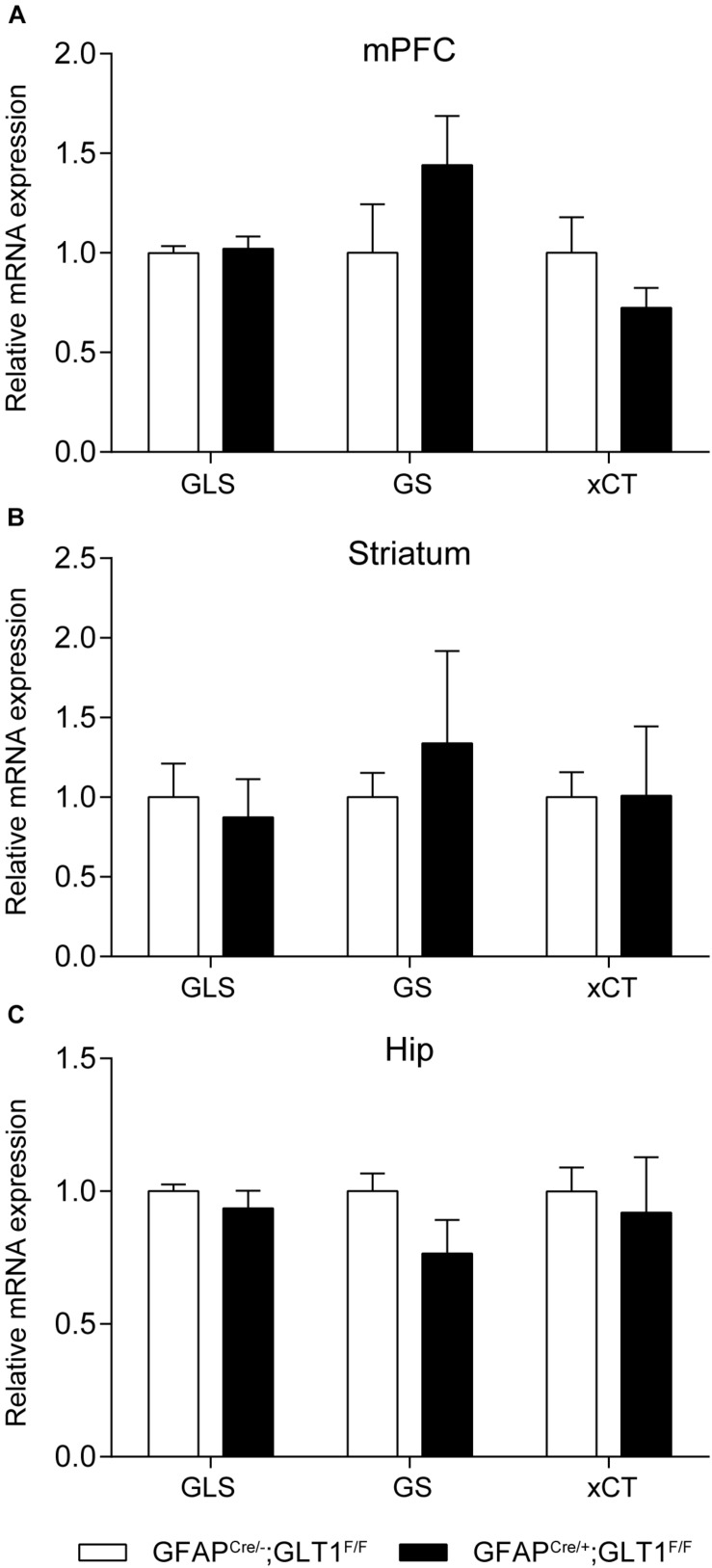
Expression of glutamate-related genes in GFAP^cre/+^; GLT1^F/F^ mice. Glutamate-related genes expression including glutaminase (GLS), glutamine synthetase (GS), Cystine/glutamate antiporter (xCT) in GFAP^cre/+^; GLT1^F/F^ mice in mPFC **(A)**, striatum **(B)**, and Hip **(C)** by Quantitative real-time PCR analysis (*n* = 3,4). The interested mRNA levels were normalized with those of GAPDH. All data are presented as mean ± SEM. Student’s *t*-tests were used to compare mRNA levels of GFAP^cre/+^; GLT1^F/F^ mice and controls for individual gene in each brain region. *p < 0.05.

In addition, we examined the expression of genes related to glutamate metabolism including glutaminase (GLS), glutamine synthetase (GS), and cystine-glutamate exchanger (xCT) in the mPFC, striatum and hippocampus brain regions by qRT-PCR. We found that the mRNA expression of these genes was similar between groups in the mPFC (two−tailed unpaired *t*-test; GLS: *t*_6_ = 0.308, *p* = 0.768; GS: *t*_6_ = 1.267, *p* = 0.252; xCT: *t*_6_ = 1.343, *p* = 0.228; Figure 2A), striatum (two−tailed unpaired *t*-test; GLS: *t*_6_ = 0.394, *p* = 0.707; GS: *t*_6_ = 0.897, *p* = 0.404; xCT: *t*_6_ = 0.018, *p* = 0.986; Figure 2B), and Hip (two−tailed unpaired *t*-test; GLS: *t*_6_ = 0.910, *p* = 0.398; GS: *t*_6_ = 1.646, *p* = 0.151; xCT: *t*_6_ = 0.355, *p* = 0.735; Figure 2C). Following this, we confirmed the protein expression of GS using Western blot since GS is used as a astrocyte marker (Anlauf and Derouiche, 2013), which also revealed no difference between groups in the mPFC, striatum and Hip (two−tailed unpaired *t*-test; mPFC: *t*_5_ = 1.148, *p* = 0.303; Striatum: *t*_5_ = 0.565, *p* = 0.596; Hip: *t*_5_ = 1.846, *p* = 0.124; Supplementary Figures S3A,B).

### GFAP-Positive Astrocytic GLT1 Deficiency Showed Decreased Anxiety- and Depression-Like Behaviors

**FIGURE 3:**
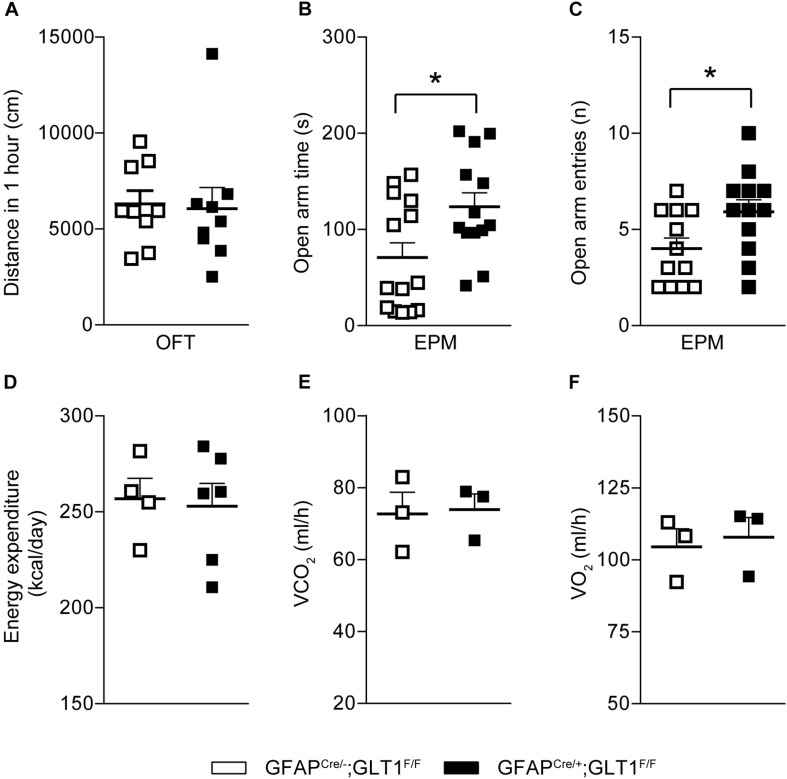
Decreased anxiety-like behaviors in GFAP^cre/+^; GLT1^F/F^ mice. **(A)** Total distance (cm) moved in the open field of the 1 h in GFAP^cre/+^; GLT1^F/F^ mice and controls (*n* = 10–14). **(B)** Time spent in open arms of GFAP^cre/+^; GLT1^F/F^ and control mice in elevated plus maze (EPM) (*n* = 13,14). **(C)** Open arm entries of GFAP^cre/+^; GLT1^F/F^ mice and controls in EPM (*n* = 13,14). **(D)** Energy expenditure of GFAP^cre/+^; GLT1^F/F^ mice and controls in the metabolic chamber (*n* = 3). **(E)** Volume of CO_2_ of GFAP^cre/+^; GLT1^F/F^ mice and controls in the metabolic chamber (*n* = 3). **(F)** Volume of O_2_ of GFAP^cre/+^; GLT1^F/F^ mice and controls in the metabolic chamber (*n* = 3). All data are presented as mean ± SEM. Statistical significance was calculated by Student’s *t*-test. *p < 0.05.

In order to investigate the basal locomotor activity, we employed the OFT. We revealed that the total distance traveled in the OFT was not different between GFAP^cre/+^; GLT1^F/F^ mice and controls (two−tailed unpaired *t*-test; *t*_16_ = 0.191, *p* = 0.851; Figure 3A). Two-way ANOVA analysis showed no significant difference between groups (two−way ANOVA; group effect: *F*_1,19_ = 0.038, *p* = 0.848; Supplementary Figure S4A). To assess the performance of astrocytic GLT1 deficient mice under stress factor, we sought to investigate the anxiety-like behaviors. In the EPM, we found that the GFAP^cre/+^; GLT1^F/F^ mice spent more time in the open arms (two−tailed unpaired *t*-test; *t*_25_ = 2.492, *p* = 0.02; Figure 3B), and entered the open arms more frequently (two−tailed unpaired *t*-test; *t*_22_ = 2.284, *p* = 0.032; Figure 3C). In addition, we showed no significant difference between GFAP^cre/+^; GLT1^F/F^ mice and controls in closed arms entries (two−tailed unpaired *t*-test; *t*_23_ = 0.105, *p* = 0.918; Supplementary Figure S4B) and total entries (two−tailed unpaired *t*-test; *t*_23_ = 1.347, *p* = 0.191; Supplementary Figure S4C). Next, we examined whether the basal metabolic activity was affected by the deletion of GLT1 in astrocytes, as assessed by the metabolic chamber for a continuous 48 h. We found that GFAP^cre/+^; GLT1^F/F^ mice exhibited normal energy expenditure (two−tailed unpaired *t*-test; *t*_8_ = 0.227, *p* = 0.826; Figure 3D), volume of CO_2_ (two−tailed unpaired *t*-test; *t*_4_ = 0.161, *p* = 0.880; Figure 3E), and volume of O_2_ (two−tailed unpaired *t*-test; *t*_4_ = 0.32, *p* = 0.736; Figure 3F) metabolism. Taken together, our results showed a decrease in anxiety-like behavior of astrocytic GLT1 deficiency without altering basal locomotion and caloric consumption.

**FIGURE 4:**
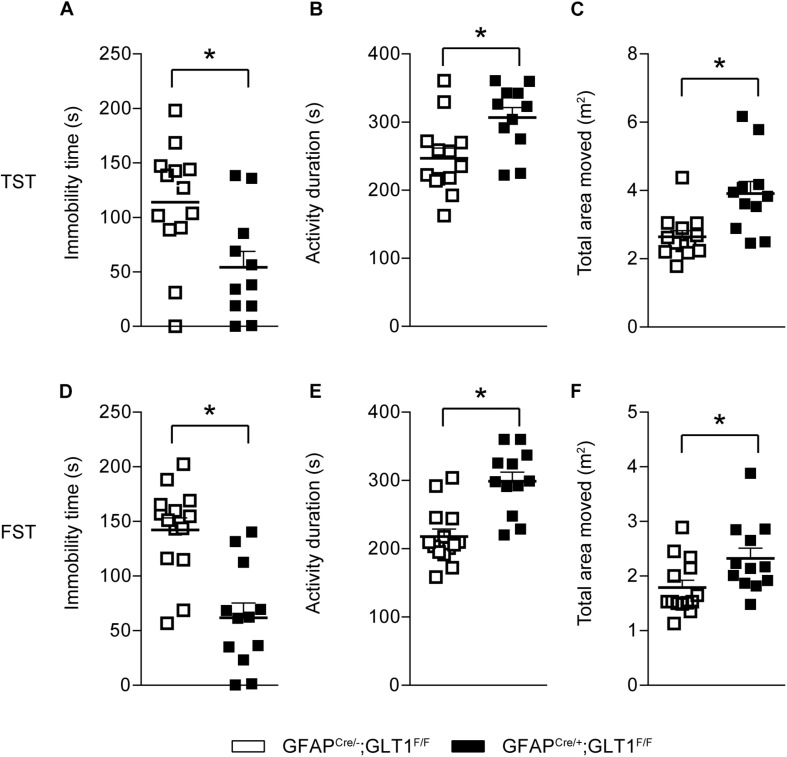
Decreased depression-like behaviors in GFAP^cre/+^; GLT1^F/F^ mice. **(A)** Immobility time of GFAP^cre/+^; GLT1^F/F^ mice and controls in tail suspension test (TST) (*n* = 12–14). **(B)** Total activity duration in TST of GFAP^cre/+^; GLT1^F/F^ and control mice (*n* = 12–14). **(C)** Total area (m^2^) moved in TST of GFAP^cre/+^; GLT1^F/F^ and control mice (*n* = 12–14). **(D)** Immobility time of GFAP^cre/+^; GLT1^F/F^ mice and controls in forced swim test (FST) (*n* = 12–14). **(E)** Total activity duration in FST of GFAP^cre/+^; GLT1^F/F^ mice and controls (*n* = 12–14). **(F)** Total area (m^2^) moved in FST of GFAP^cre/+^; GLT1^F/F^ mice and controls (*n* = 12–14). All data are presented as mean ± SEM. Statistical significance was calculated by Student’s *t*-test. *p < 0.05.

Additionally, in the tail suspension test (TST), GFAP^cre/+^; GLT1^F/F^ mice showed less immobile time (two−tailed unpaired *t*-test; *t*_22_ = 2.823, *p* = 0.01; Figure 4A), had a longer activity duration (two−tailed unpaired *t*-test; *t*_22_ = 2.82, *p* = 01; Figure 4B), and also moved a greater area (two−tailed unpaired *t*-test; *t*_22_ = 3.319, *p* = 0.003; Figure 4C) compared to controls. Similarly, in the forced swim tests (FSTs), GFAP^cre/+^; GLT1^F/F^ mice exhibited less immobile time (two−tailed unpaired *t*-test; *t*_24_ = 4.668, *p* < 0.0001; Figure 4D), had a longer activity duration (two−tailed unpaired *t*-test; *t*_24_ = 4.691, *p* < 0.0001; Figure 4E), and also moved a greater area (two−tailed unpaired *t*-test; *t*_24_ = 2.379, *p* = 0.026; Figure 4F) compared to controls. Therefore, we conclude that reduced anxiety- or depression-like behaviors were found in GFAP^cre/+^; GLT1^F/F^ mice.

### Reduced Freezing Response in the Fear Contextual and Cued Tests of GFAP-Positive Astrocytic GLT1 Deficient Mice

**FIGURE 5:**
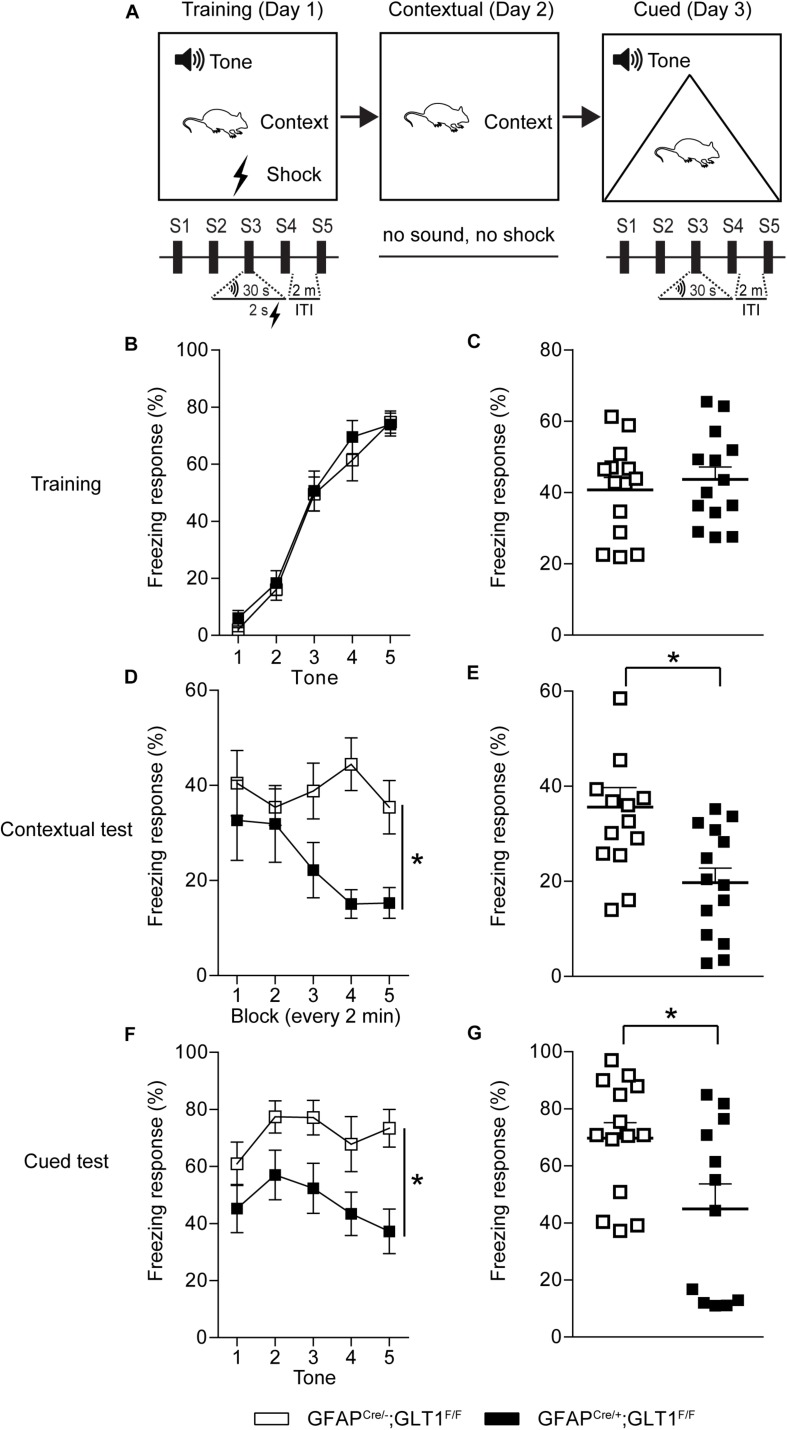
Reduced freezing time of GFAP^cre/+^; GLT1^F/F^ mice in contextual and cued fear memory tests. **(A)** The experimental schedule for performing fear conditioning test. **(B)** The learning curve of fear conditioning showed the freezing response for each tone of GFAP^cre/+^; GLT1^F/F^ mice and controls during the training day 1 (*n* = 14). **(C)** The total freezing response of GFAP^cre/+^; GLT1^F/F^ mice and controls during the training day 1 (*n* = 10–14). **(D)** The curve of fear conditioning showed the freezing response for each block (every 2 min) of GFAP^cre/+^; GLT1^F/F^ mice and controls on day 2 during the contextual test (*n* = 14). **(E)** The freezing response of GFAP^cre/+^; GLT1^F/F^ mice and controls during the contextual test day 2 (*n* = 14). **(F)** The curve of fear conditioning showed the freezing response for each tone of GFAP^cre/+^; GLT1^F/F^ mice and controls on day 3 during the cued test (*n* = 14). **(G)** The freezing response of GFAP^cre/+^; GLT1^F/F^ mice and controls during cued test day 3 (*n* = 12–14). All data are presented as mean ± SEM. Statistical significance was calculated by Student’s *t*-test in (C, E, and G), and by two-way repeated measures ANOVA with *post hoc t*-test for multiple comparisons in (B, D, and F). *p < 0.05.

To examine the role of GFAP-positive astrocytic GLT1 in anxiety-related fear, we trained mice with five tone and foot shock pairing on day 1, and then we performed the contextual and cued fear memory tests on day 2 and day 3, respectively (Figure 5A). We revealed that GFAP^cre/+^; GLT1^F/F^ mice had comparable freezing responses across the training trials (Two way ANOVA; Interaction: *F*_4,__26_ = 0.368, *p* = 0.831; Figure 5B. Two−tailed unpaired *t*-test; *t*_26_ = 0.599, *p* = 0.554; Figure 5C). Interestingly, in contextual test, we found significantly decreased freezing response in GFAP^cre/+^; GLT1^F/F^ mice compared to that of control mice (Two way ANOVA; group effect: *F*_1,26_ = 9.265, *p* = 0.005; Figure 5D. Two−tailed unpaired *t*-test; *t*_26_ = 3.099, *p* = 0.005; Figure 5E). Similarly, in cued test, GFAP^cre/+^; GLT1^F/F^ mice showed less freezing response compared to control (Two way ANOVA; group effect: *F*_1,24_ = 5.771, *p* = 0.024; Figure 5F. Two−tailed unpaired *t*-test; *t*_24_ = 2.472, *p* = 0.021; Figure 5G). These results suggested that the deletion of astrocytic GLT1 decreased response to fear memory without affecting fear memory acquisition, which may be associated with reduced anxiety.

## Discussion

This study demonstrates that GFAP-positive astrocyte-specific deletion of GLT1 reduced anxiety- and depression-like behaviors using a comprehensive array of behavioral tests including EPM, TST and FST. In addition, the freezing response in fear conditioning test was also reduced in GFAP-positive astrocytic GLT1 deficient mice. These results suggest that GFAP-positive astrocytic GLT1 is associated with emotional regulation such as anxiety, depression, and fear expression.

Previous studies have also demonstrated the association of GLT1 with emotional regulation including depression and anxiety. Chronic unpredictable stress associated with depressive behaviors, was found to decrease hippocampal GLT1 protein (Zhu et al., 2017). In stressed rodents, reduced GLT1 protein was found in the PFC of females, while reduced GLT1 was found in the striatum of males (Rappeneau et al., 2016). Hippocampal glial numbers show sex differences as well, and are reduced with prenatal stress and associated with emotional regulation and cognitive function in humans and rodents (Behan et al., 2011; Blacker et al., 2019). These studies together showed a reduction of GLT1 in PFC, striatum, and hippocampus associated with depressed status complicated by sex factors too. The administration of GLT1 selective inhibitor DHK induces robust antidepressant-like responses (Gasull-Camos et al., 2017), which is consistent with our findings of tail suspension and FSTs with the astrocytic GLT1 KO mice displaying a shorter immobile time. However, administration of DHK in the central amygdala induced depression and anxiety (John et al., 2015). Similarly, the deletion of GLT1 in habenula astrocytes was found to exacerbate depression-like behaviors (Cui et al., 2014). These studies suggest behavioral effects different from global GLT1 inhibition, which is expected since multiple brain regions and their interactions regulate depression and anxiety behaviors.

In addition, global GLT1 KO mice exhibited severe seizure activity with lethal spontaneous seizures and high glutamate levels (Tanaka et al., 1997). Petr et al. (2015) reported that only astrocytic but not neuronal deletion of GLT1 induced seizure activity, suggesting that the function of an astrocytic membrane protein is particularly important in the pathophysiology of multiple behaviors related to glutamatergic system. Furthermore, brain-region-specific deletion of GLT1 in the diencephalon, brainstem, and spinal cord could result in excess mortality and lethal spontaneous seizure (Sugimoto et al., 2018). Likewise, GLT1 dysfunction in the dorsal forebrain is involved in the pathogenesis of infantile epilepsy (Sugimoto et al., 2018). Overall, brain regional GLT1 plays individual roles, respectively, which might be an important future study direction.

As reported, we also observed a severe seizure phenotype for some mice starting around 3 months of age. Thus, to avoid confounding effects on our behavioral analysis, we exclude those mice showing seizure behaviors. The early stage of spontaneous seizures behavior in mice including grooming, jumping, and nodding etc. can be recognized as a type of repetitive behavior, which is highly correlated with anxiety (Crawley, 2007). It is believed that a slight elevation to glutamate levels may promote repetitive behaviors, whereas greater glutamate elevations more readily induce stereotypies and limbic seizure behaviors (McGrath et al., 2000; Chakrabarty et al., 2005).

In human studies, patients with anxiety and/or depression displayed enhanced fear memory and extinction (Cuthbert et al., 2003; Dibbets et al., 2015). In this study, astrocytic GLT1 deficiency did not affect the fear memory acquisition, suggesting that mice were able to adapt to the potential dangerous stimuli. In both contextual and cued fear memory tests, astrocytic GLT1 deficient mice showed less freezing response, indicating less fear toward the stimulus, which was also reflected by less anxiety-like behaviors in the EPM of astrocytic GLT1 deficient mice. Notably, similar decrement of anxiety was also found in a SPRED2-deficient mouse model of obsessive compulsive disorder (OCD) (Ullrich et al., 2018). However, the reduced anxiety-like behaviors in OCD mice is controversial to traditional knowledge that anxiety or depression is commonly comorbid with OCD. Interestingly, previous investigations reported that astrocytic GLT1 deficiency also showed excessive repetitive behaviors in astrocytic GLT1 deficiency, which might be caused by glutamatergic hyperactivity (Aida et al., 2015; Katz et al., 2019) since the high glutamate level was found in mice with both global and astrocytic deletion of GLT1 (Tanaka et al., 1997; Petr et al., 2015). In current study, we also do not exclude a possibility that GLT1 deletion in astrocyte might induce repetitive behavior since we saw the slightly increased locomotor activity in first 10 min of the OFT, and also the total entries in the EPM, which might be a sign of that they were repetitively checking the new environment. Overall, we concluded that the less anxious and excessive repetitive behavior might be revealed in GFAP-positive astrocytic GLT1 deficient mice. However, the contribution of GLT1 in regard to OCD, anxiety, and depressive behavior remains highly controversial.

In summary, this investigation suggests that GFAP-positive astrocyte-specific deletion of GLT1 decreased anxiety- and depression-like behaviors, which may provide novel insights toward the underlying mechanisms of mood related psychiatric disorders.

## Data Availability Statement

The datasets generated for this study are available on request to the corresponding author.

## Ethics Statement

The animal study was reviewed and approved by the Mayo Clinic Institutional Animal Care and Use Committee.

## Author Contributions

D-SC was responsible for the study concept and design and drafted the manuscript. Y-FJ performed all the data analysis, also helped draft the manuscript and interpret the findings. Y-FJ and KW contributed to the acquisition of animal data. AH and LP assisted with the data analysis and interpretation of findings. Y-FJ, KW, AH, LP, MB, and D-SC provided critical revision of the manuscript for important intellectual content. All authors critically reviewed content and approved final version for publication.

## Conflict of Interest

D-SC is a scientific advisory board member to Peptron Inc. and Peptron Inc. had no role in the preparation, review, or approval of the manuscript; nor the decision to submit the manuscript for publication. The remaining authors declare no biomedical financial interests or potential conflicts of interest.

## Supplementary Material

FIGURE S1 The specificity of GFAP-Cre line. **(A)** GLT1 immunohistochemistry for three brain regions including the mPFC, striatum, and hippocampus. Higher magnification of images showed from white boxes in **(B)**. Scale bar = 20 μm. **(B)** A higher magnification of GLT1 immunohistochemistry for three brain regions including the mPFC, striatum, and hippocampus. Scale bar = 10 μm.

FIGURE S2 The specificity of GFAP-Cre line. **(A)** The mapping of GFAP-Cre-positive cells in the striatum using astrocytic (anti-GFAP antibody), **(B)** microglia (anti-Iba1 antibody), **(C)** and neuronal (anti-NeuN antibody) markers, respectively. White arrows indicate the typical co-localization or no co-localization. Scale bar = 50 μm.

FIGURE S3 The protein expression of GS in GFAP^cre/+^; GLT1^F/F^ mice. **(A)** Western blot analysis of GS in the mPFC, striatum, and Hippocampus. GS band intensities were normalized with those of GAPDH. **(B)** The quantitative analysis of western blot of **(A)** (*n* = 3,4). All data are presented as mean ± SEM. Statistical significance was calculated by Student’s *t*-test in **(B)**.

FIGURE S4 No difference in total locomotion of GFAP^cre/+^; GLT1^F/F^ mice in open field and elevated plus maze tests. **(A)** Distance moved (cm) in the open field of every 10 min in GFAP^cre/+^; GLT1^F/F^ mice and controls (*n* = 9,12). **(B)** Closed arm entries of GFAP^cre/+^; GLT1^F/F^ mice and controls in EPM (*n* = 12,13). **(C)** Total entries of GFAP^cre/+^; GLT1^F/F^ mice and controls in EPM (*n* = 12,13). All data are presented as mean ± SEM. Statistical significance was calculated by Student’s *t*-test in **(B,C)**, and by two-way repeated measures ANOVA with *post hoc t*-test for multiple comparisons in **(A)**.
