# Supplementary figures and images for: Astrocytic Glutamate Transporter 1 (GLT1) Deficiency Reduces Anxiety- and Depression-Like Behaviors in Mice
Source: Front Behav Neurosci. 2020 Apr 22;14:57. doi: 10.3389/fnbeh.2020.00057 (PMC7189218; doi:10.3389/fnbeh.2020.00057)

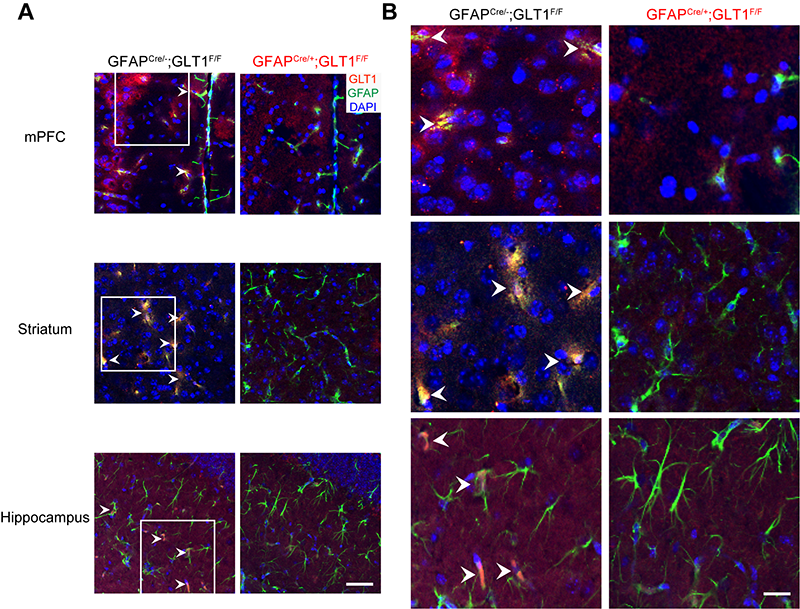

Supplement: FIGURE S1 — The specificity of GFAP-Cre line. (A) GLT1 immunohistochemistry for three brain regions including the mPFC, striatum, and hippocampus. Higher magnification of images showed from white boxes in (B). Scale bar = 20 μm. (B) A higher magnification of GLT1 immunohistochemistry for three brain regions including the mPFC, striatum, and hippocampus. Scale bar = 10 μm. [file Image_1.TIF]

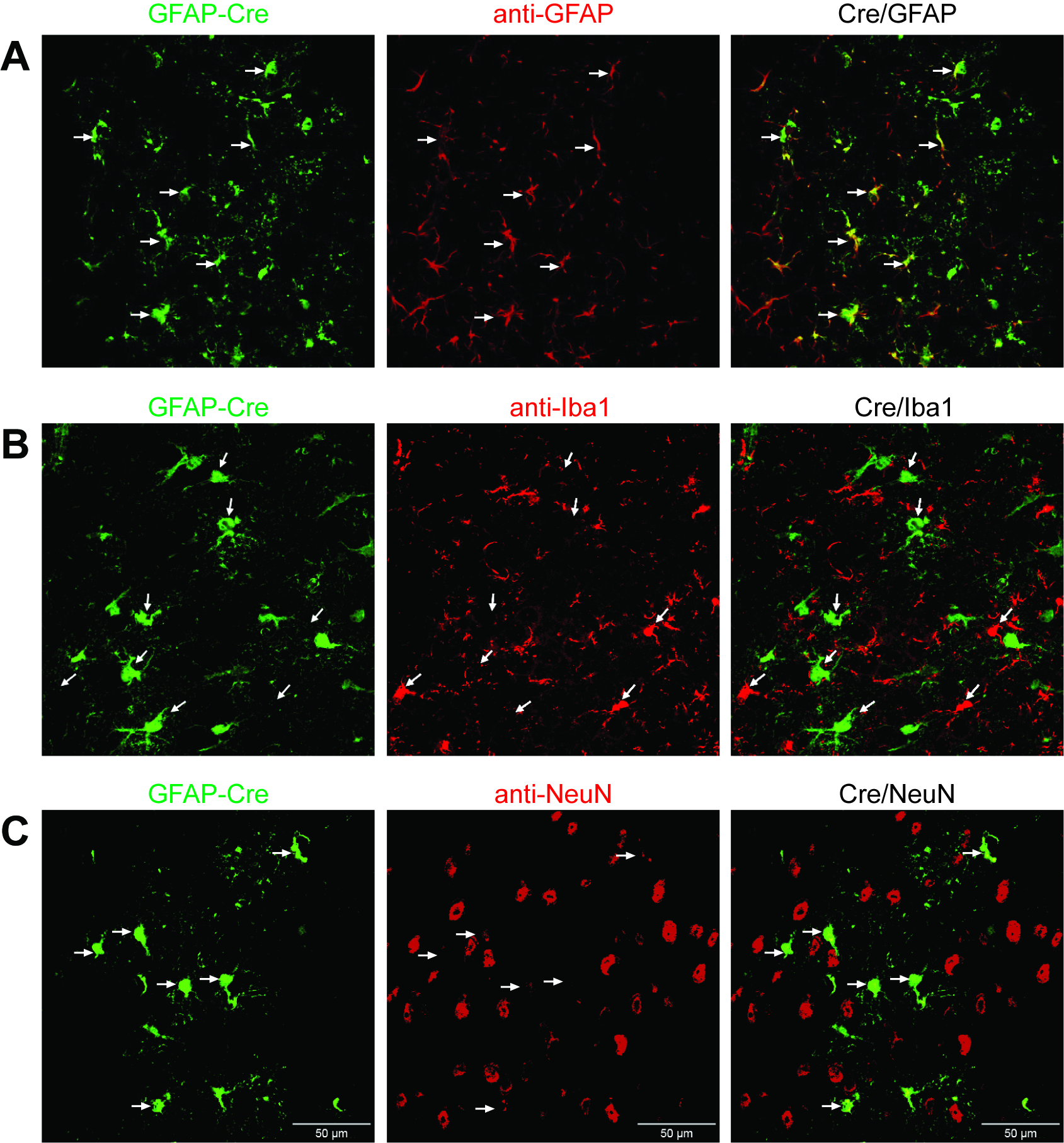

Supplement: FIGURE S2 — The specificity of GFAP-Cre line. (A) The mapping of GFAP-Cre-positive cells in the striatum using astrocytic (anti-GFAP antibody), (B) microglia (anti-Iba1 antibody), (C) and neuronal (anti-NeuN antibody) markers, respectively. White arrows indicate the typical co-localization or no co-localization. Scale bar = 50 μm. [file Image_2.TIF]

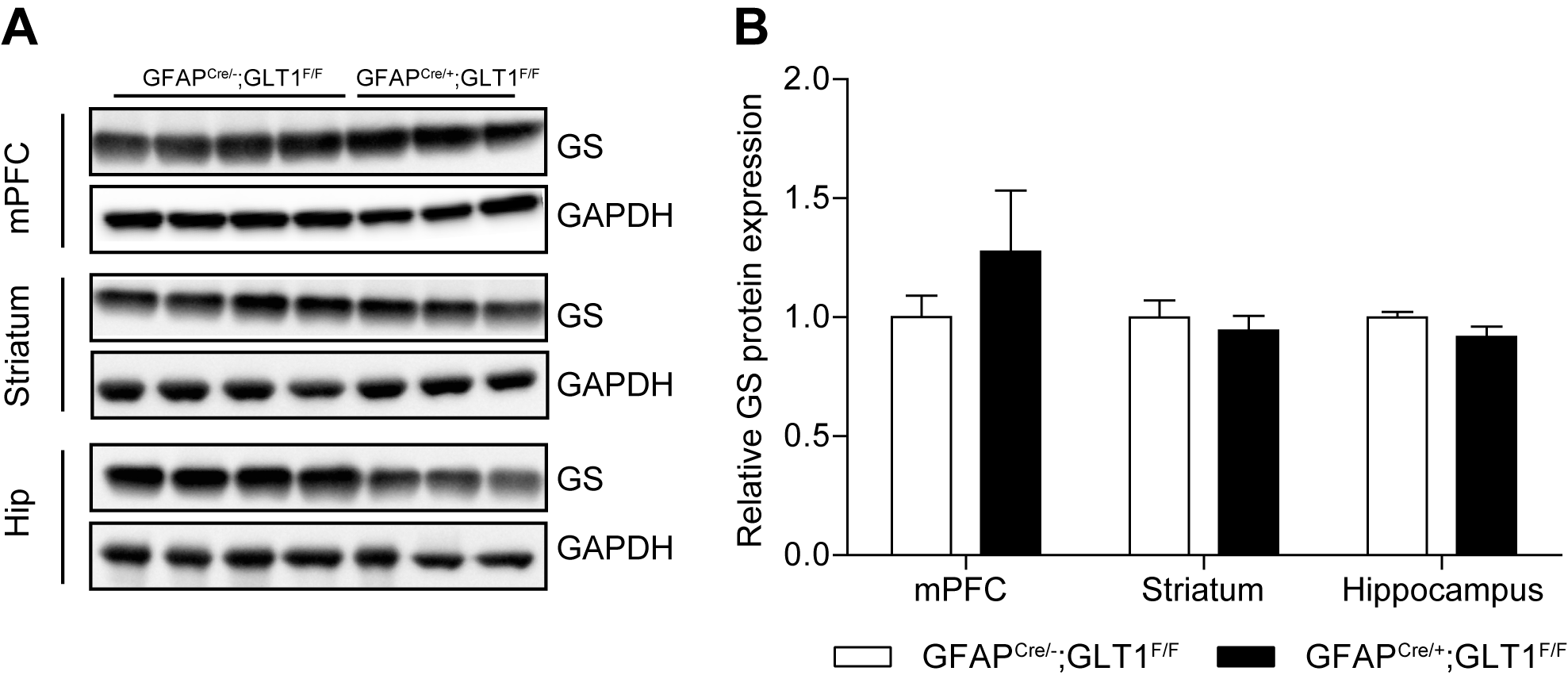

Supplement: FIGURE S3 — The protein expression of GS in GFAPcre/+; GLT1F/F mice. (A) Western blot analysis of GS in the mPFC, striatum, and Hippocampus. GS band intensities were normalized with those of GAPDH. (B) The quantitative analysis of western blot of (A) (n = 3,4). All data are presented as mean ± SEM. Statistical significance was calculated by Student’s t-test in (B). [file Image_3.TIF]

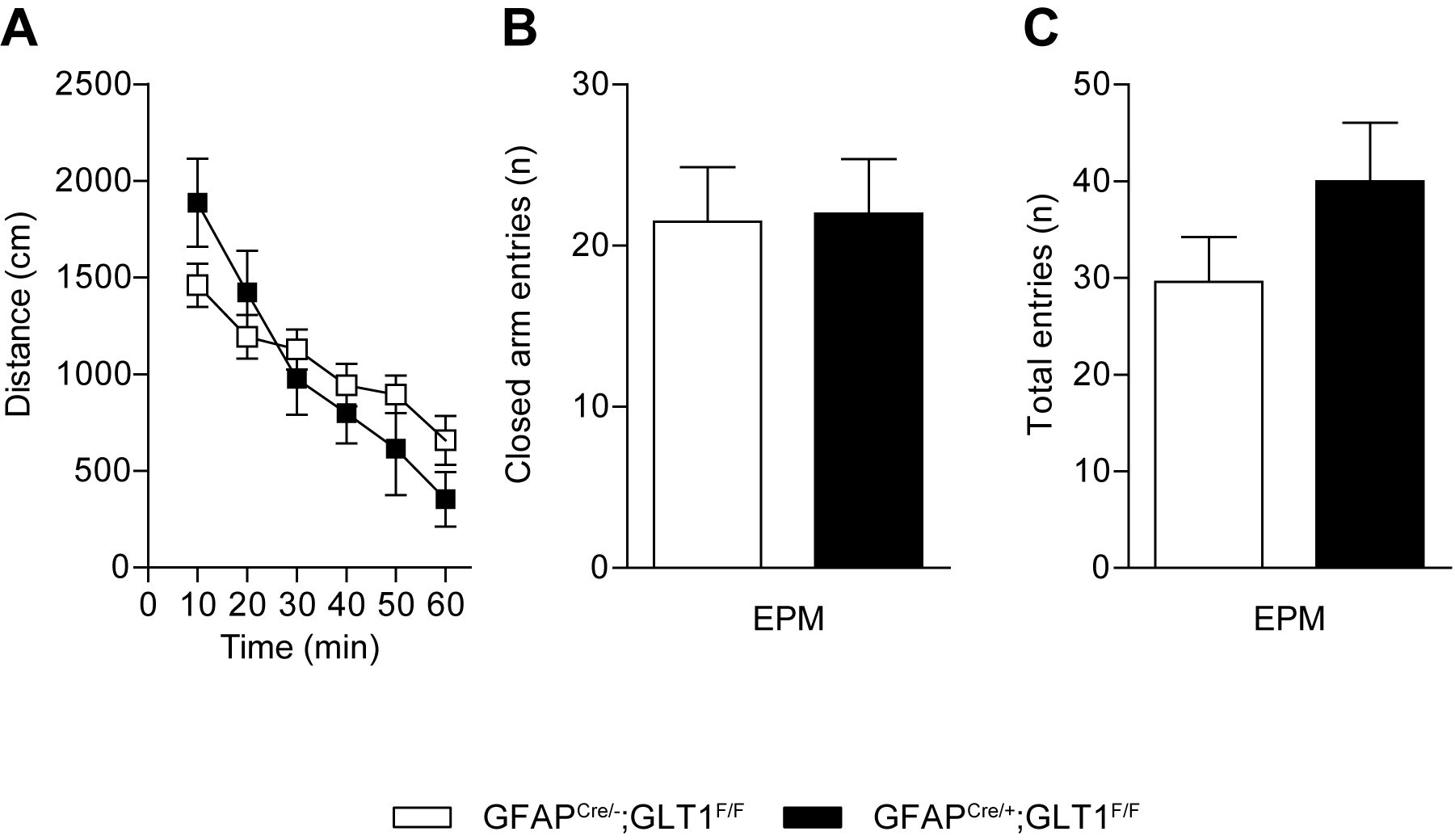

Supplement: FIGURE S4 — No difference in total locomotion of GFAPcre/+; GLT1F/F mice in open field and elevated plus maze tests. (A) Distance moved (cm) in the open field of every 10 min in GFAPcre/+; GLT1F/F mice and controls (n = 9,12). (B) Closed arm entries of GFAPcre/+; GLT1F/F mice and controls in EPM (n = 12,13). (C) Total entries of GFAPcre/+; GLT1F/F mice and controls in EPM (n = 12,13). All data are presented as mean ± SEM. Statistical significance was calculated by Student’s t-test in (B,C), and by two-way repeated measures ANOVA with post hoc t-test for multiple comparisons in (A). [file Image_4.TIF]
